# Immunomodulating and Revascularizing Activity of *Kalanchoe pinnata* Synergize with Fungicide Activity of Biogenic Peptide Cecropin P1

**DOI:** 10.1155/2017/3940743

**Published:** 2017-06-11

**Authors:** N. S. Zakharchenko, A. S. Belous, Y. K. Biryukova, O. A. Medvedeva, A. V. Belyakova, G. A. Masgutova, E. V. Trubnikova, Y. I. Buryanov, A. A. Lebedeva

**Affiliations:** ^1^Branch of Shemyakin-Ovchinnikov Institute of Bioorganic Сhemistry, Russian Academy of Sciences, Science Avenue 6, Pushchino, Moscow Region 142290, Russia; ^2^Kursk State Medical University, K. Marksa St 3, Kursk 305041, Russia; ^3^Kursk State University, Radisheva St 33, Kursk 305000, Russia; ^4^Emanuel Institute of Biochemical Physics, Kosygina St 4, Moscow 119334, Russia; ^5^Chumakov Institute of Poliomyelitis and Viral Encephalitides, 27 km Kievskogo shosse, Moscow 142782, Russia; ^6^Kazan Federal University, Kremlevskaya St 18, Kazan 420008, Russia

## Abstract

Previously transgenic *Kalanchoe pinnata* plants producing an antimicrobial peptide cecropin P1 (CecP1) have been reported. Now we report biological testing *K. pinnata* extracts containing CecP1 as a candidate drug for treatment of wounds infected with *Candida albicans.* The drug constitutes the whole juice from *K. pinnata* leaves (not ethanol extract) sterilized with nanofiltration. A microbicide activity of CecP1 against an animal fungal pathogen in vivo was demonstrated for the first time. However, a favorable therapeutic effect of the transgenic *K. pinnata* extract was attributed to a synergism between the fungicide activity of CecP1 and wound healing (antiscar), revascularizing, and immunomodulating effect of natural biologically active components of *K. pinnata*. A commercial fungicide preparation clotrimazole eliminated *C. albicans* cells within infected wounds in rats with efficiency comparable to CecP1-enriched *K. pinnata* extract. But in contrast to *K. pinnata* extract, clotrimazole did not exhibit neither wound healing activity nor remodeling of the scar matrix. Taken together, our results allow assumption that CecP1-enriched *K. pinnata* extracts should be considered as a candidate drug for treatment of dermatomycoses, wounds infected with fungi, and bedsores.

## 1. Introduction

*Kalanchoe pinnata* (*Bryophyllum pinnatum*) (Lamarck) Persoon (Crassulaceae) is a succulent perennial plant native to Madagascar. It is used for healing wounds in a traditional medicine to treat psychiatric disorders and as a tocolytic agent to prevent premature labour [1]. Extracts of *K. pinnata* and some other Kalanchoe species (*K. crenata*, *K. brasiliensis*, and *K. daigremontiana*) are reported to exhibit antimicrobial [2, 3] and virucide [4–6] activity. Further, they were found to kill leishmanias [7] and malaria plasmodium [8]. It induces the relaxation of smooth muscles [9]; exhibits antimutagenesis, antihistamine [10], and hepatoprotective activity [11]; causes immunomodulatory and anti-inflammatory effects [12, 13]; and inhibits thyroid peroxidase [14]. Baginskaya and Leskova [15] reported a positive effect of *K. pinnata* extract towards experimental gastric ulcer in mice. Eventually, it demonstrates an oncolytic activity on certain models [16]. To our best knowledge, primary experimental data about testing antifungal activity of Kalanchoe extracts have not been published, but occurrence of this effect is mentioned in a review by Kutsik and Zuzuk [17].

Like other species of the genus *Kalanchoe*, *K. pinnata* contains a number of biologically active compounds which may contribute to pharmacological properties of its extracts:

1. Flavonoid glycosides: two phenolic glucosides, syringic acid *β*-D-glucopyranosyl ester and 4′-O-*β*-D-glucopyranosyl-cis-p-coumaric acid; nine flavonoids including kaempferol, quercetin, myricetin, acacetin, and diosmetin glycosides; and flavonol glycosides: quercetin (3-O-*α*-L-arabinopyranosyl-(1 → 2)-*α*-L-rhamnopyranoside 7-O-*β*-D-glucopyranoside) and myricetin (3-O-*α*-L-arabinopyranosyl-(1 → 2)-*α*-L-rhamnopyranoside) [18].
2. Four bufadienolides: bersaldegenin-1-acetate, bryophyllin A, bersaldegenin-3-acetate, and bersaldegenin-1,3,5-orthoacetate [18].
3. Blood-agglutinating lectins with Mr 44–47 kDa containing ~1.5% carbohydrate [19, 20].

Over the last decade, ethanol extracts became the most popular form of Kalanchoe medicinal application [21–23]. It means that lipophilic constituents of the fresh juice (bufadienolides, polyphenols, and flavonoids) are suggested to confer essential part of their total activity. However, an evident biological effect of Kalanchoe lectins has been formerly documented. Lapchik et al. demonstrated a high mitogenic and blast-transforming activity of lectins from different Kalanchoe species, particularly from *K. blossfeldiana* [24]. A study of lectins from 52 Kalanchoe species cultivated in Kiev State University's botanical garden in 1982 demonstrated a drastic difference in their virucide activity. For instance, *K. daigremontianum* broadly used in pharmacy did not exhibit virucide activity. In contrast, *K. velutina*, *K. blossfeldiana*, *K. pinnata*, and *K. crenata* demonstrated a high neutralizing activity in vitro towards nonenveloped RNA-containing viruses (vaccine poliovirus Sabin type II, Coxsackie B1, and Coxsackie B6). 50% reduction of the virus titter was achieved after incubation of these viruses with fresh juice of *K. velutina* diluted with cultural medium in ratio 1 : 32768, and *K. pinnata*, *K. blossfeldiana*, and *K. crenata* diluted 1 : 16384–1 : 8192 times. An enveloped influenza virus A (Hong Kong) 1/68/H3N2 could be 50% inactivated by a juice of *K. velutina* in dilution 1 : 400, by juice of *K. crenata*—in dilution 1 : 200, and *K. pinnata*, *K. blossfeldiana*, and *K. beharensis*—in dilution 1 : 100 [20]. The rate of the virus neutralization did not depend on the temperature. Electronic microscopy demonstrated the contact of the viral particles with Kalanchoe juice induced their deformation and aggregation. Taken together, these observations may be explained by agglutination of the viral particles by Kalanchoe lectins or blocking their cell-specific receptors.

Hence, lectins may contribute to both antipathogenic and immunomodulatory activities of Kalanchoe extracts by binding carbohydrate moieties of extracellular receptors. Immunomodulatory functions may be mediated by nonproteinaceous components of these extracts as well. Costa et al. [25] reported inhibition of human lymphocyte proliferation with patuletin acetylrhamnosides from *K. brasiliensis*. Da-Silva et al. [26] communicated about the induction of Th1-mediators (IL-2 and IFN-*γ*) and suppression of Th2-mediator IL-4 in mice after the administration of *K. brasiliensis* ethanol extract.

Recently, transgenic plants of *K. pinnata* producing antimicrobial peptide (AMP) cecropin P1 (CecP1) from *Ascaris suis* was described [27]. An antimicrobial, antifungal, and antiviral activity was attributed to this AMP [28]. However, data about biological trials of CecP1 towards human or animal microbial pathogens are scarce due to poor availability of the synthetic peptide and difficulty of its biogenic synthesis in bacteria or yeast which suffer from its toxicity [29].

This work pursues testing antifungal and wound healing activity of *K. pinnata* plants producing CecP1 towards highly prevalent human fungal pathogen *Candida albicans* on a model of infected wounds in rats. Taking into account the probability of existence of an intrinsic antifungal activity in *K. pinnata* juice (although not formerly reported), a juice of nontransgenic *K. pinnata* was tested in parallel as a negative control. Since CecP1 is not soluble in ethanol, a water extract of the plants was tested. In this respect, occurrence of lectins exhibiting lymphoproliferative and immunomodulatory properties along with bufadienolides and flavonoids in this type of preparations may be noteworthy. A commonly used commercial medical fungicide clotrimazole was used as a positive control (standard).

## 2. Methods

### 2.1. Producing *K. pinnata* Extracts

*K. pinnata* extracts were prepared form leaves of *K. pinnata* transgenic plants bearing a binary vector pBM-cecP1 for *Agrobacterium tumefaciens* vector with T-element randomly integrated to a plant genome. The vector was free from drug-resistance markers. The transgenic plants were selected after Agrobacterium-mediated transformation by a direct immunological testing exhibited a steady yield of CecP1 for at least two years as checked by three independent methods (immunoblotting, plate test for microbicide activity, and HPLC combined with mass-spectroscopy detection). 5.07 L of extract was produced from 3 kg of the recombinant plant leaves using deionized water as a solvent. After heating the fresh extract at +80°C and its sterilizing filtration through a nylon membrane with 0.22 *μ*m pores, the extracts were adjusted to a total protein concentration ~1 mg/ml. Usually, they contained 0.69–0.82 *μ*g/ml CecP1. The extracts of the wild-type (nonrecombinent) *K. pinnata* was produced by the same method and adjusted to the same concentration of the total protein.

### 2.2. Animals

In vivo trial was designed according to the European Convention about defense of the vertebrates used for experiments or for another scientific aims (Strasbourg, Mar. 18, 1986) of ETS N123. In total, 120 adult male Wistar rats weighing 180 ± 20.0 g 3-4 months old were allocated for the experiment. After quarantine, they were kept in individual cages. All animals were contained in equal terms on a standard diet and photoperiod (twelve hours of darkness and twelve hours of light). They had a ready access to water and food.

The animals were randomly divided into experimental, positive control, reference, and negative control groups (30 animals per each). All 120 animals after wounding were infected with *C. albicans*. The experimental group was treated with CecP1-enriched recombinant *K. pinnata* extract (*K. pinnata* + CecP1). The positive control group was treated with clotrimazole. The negative control group was subjected to mock treatment with a saline, and the reference group was treated with wild-type *K. pinnata* extract. Each group was divided into three echelons withdrawn from the experiment at 3th, 10th, and 14th day after the beginning of the curing.

### 2.3. Fungal Pathogen Strain

The human fungal pathogen *C. albicans* (NCTC 2625) formerly isolated from a human clinical specimens was purchased from a type strain collection of Tarasevich Research Institute for Standardization and Control of Medical Biological Preparations. *C. albicans* culture was obtained by cultivation on a slant meat-peptone nutrient agar supplemented with 1% glucose for at 37°C for 18–20 h. The cells were washed from the slant agar with a sterile saline, thoroughly suspended, adjusted to a concentration ~10^9^ CFU per ml by using an optical turbidity standard CCA 42-28-29-85 and used for infecting the wounds in rats.

### 2.4. Surgical Manipulations, Treatment, and Planimetry Assay of the Wounds

A purulent infection of wounds was modeled in rats using a method described previously [30]. The animals were anesthetized with diethyl ether. A square shape 20 × 20 mm skin area on the back of each animal was thoroughly shaved, treated by a disinfectant (70% ethanol), and then derma and epidermis were surgically removed. 1 ml of the yeast suspension containing 10^9^ CFU/ml *C. albicans* NCTC 2625 was distributed over the surface of the wound. For standardizing the wound healing conditions, the wound cavity was closed with a gauze bandage coupled to the skin.

In 36 h after wounding and infection, all animals exhibited clear symptoms of suppuration and inflammation. In this moment, the stitches and the bandage were removed, and the wound cavity was thoroughly washed from the pus. The initial wound area was determined by its lineation at a sterile transparent polyethylene film. Then the wounds were treated with 3% hydrogen peroxide as described by [31] and subjected to a specific treatment. The described medical procedure was repeated daily for 14 days after the beginning of the curing.

The negative control group was treated with 3% hydrogen peroxide and the mock medicine (a sterile saline). Other groups were treated in the same way using 10% clotrimazole or undiluted *K. pinnata* extract containing 1 mg/ml total protein (experimental preparation contained 0.7 *μ*g/ml CecP1) instead of the saline.

The animals were examined daily, and stages of wound healing (inflammation, granulation, and maturation (marginal epithelization)) were fixed.

The planimetric analysis of the wound recovery percentage was carried out at 3th, 10th, and 14th day after the beginning of curing. After this, one echelon (10 animals from each group) was withdrawn from the experiment. The animals were sacrificed with overdose of the ether anesthesia.

Residual wound square was measured individually in each animal as described formerly [32], using an imprint on a transparent polyethylene sheet which was scanned at resolution 200 pcs/inch using a common office scanner. The images were acquired in a format Adobe Photoshop CS5 Extended. The object was selected using the abovementioned standard software, and its square was automatically calculated by selecting a menu command “Analysis.” An average mean and a standard deviation (M ± Std. Dev.) were calculated for each group/echelon (10 animals). The recovery percentage was evaluated with following formula:

Recovery percentage = (initial wound square − wound square on the day *X*) × 100/(initial wound surface), where *X* = the day of wound square measurement (0 = beginning of the curing).

### 2.5. Preparing Histological Slides, Their Staining, and Analysis

After the completion of treatment, animals were sacrificed, and tissue samples were prepared as described previously [32]. The resulting biological material was fixed in 10% neutral formalin. After fixation, the tissues dissected for 1 × 1 cm fragments, washed, dehydrated, and impregnated with paraffin by standard methods. 5–7 *μ*m thick microtome sections were stained with hematoxylin and eosin to assess the density of collagen fibers. A light microscope Leica CME with magnifications ×40, ×100, ×200, and ×400 and camera DCM-510 were used. Ten sections from each wound were investigated and evaluated. Image tools 3 software (UTHSCSA ImageTool) was used for accumulating and analyzing the images.

Microscopy and photographing the slides were performed using an optical system by Leica comprising an eyepiece camera and software for documenting images FUTURE WINJOE (supplied by the manufacturer of the hardware). Cell content nearby the wound edge and underlying tissue were examined at each micrograph. The cells were categorized into neutrophils, macrophages, mast cells, fibroblasts, and capillary endotheliocytes according to their morphological features (shape of the cell, shape of the nucleus, and cytoplasm/nucleus ratio) as described previously [33]. Regularity of the collagen fiber alignment was evaluated. The percentage of those representatives of a cell population was calculated after counting 100 cells in several nonoverlapping visual fields (at least 10).

### 2.6. Microbiological Analysis of the Wound Bed Microbial Contamination

App. 0,5 g tissue (fibrous mass, infiltrate, and underlying derma) was sampled from each sacrificed animal under aseptic conditions, weighed at analytical grade balances (accuracy 0.1 mg), placed into a sterile porcelain mortar, mixed with a sterile saline in a weight ratio 1 : 10, and homogenized with a sterile pestle for 3 min at room temperature. The homogenate was diluted 1000 times with a sterile saline (with three consequent steps 1 : 10 using 1 mL samples) and 100 *μ*l aliquot of each dilution was inoculated to Petri dishes with meat-peptone nutrient agar supplemented with 0.1% glucose. The inoculated dishes were incubated at 37 ± 1°C for 72 h and then 1 day more at a room temperature. The colonies were counted and number of CFU recalculated per 1 g tissue. The count was suggested to be valid if number of colonies was between 30 and 300 per dish.

### 2.7. Statistical Analysis

Statistical analysis was provided by Microsoft Excel 2007 and program “Statistics” 8.0 StatSoft. The average values and standard deviations of quantitative indexes obtained by planimetry and microbiological methods were calculated for each group/echelon (10 animals). The statistical significance of differences between the groups/echelons was estimated by the Mann–Whitney *U* test (*p* < 0.05).

## 3. Results

### 3.1. Study of the Wound Healing Activity by Planimetry

**Figure 1:**
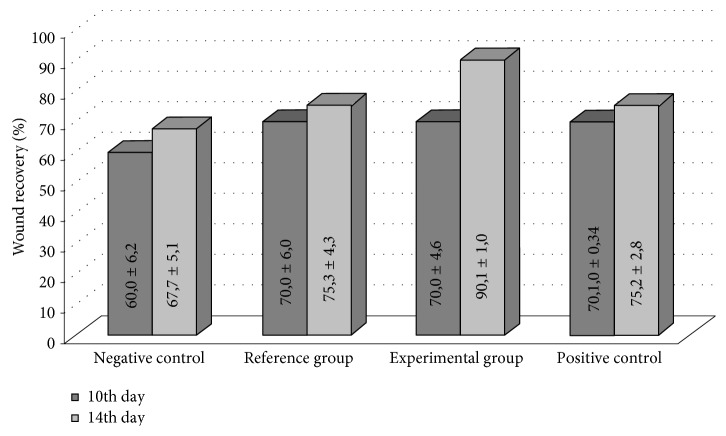
Planimetry examination of healing of the wounds in rats infected with *C. albicans.* Wound recovery percentage at 10th and 14th days after beginning of curing is shown (M ± Std. Dev.).

**Figure 2:**
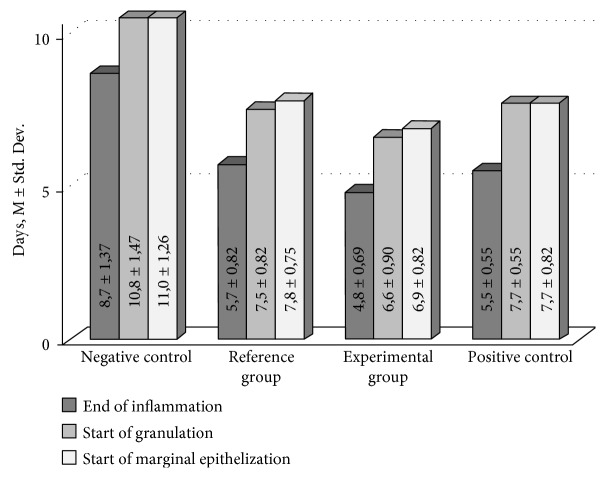
Duration of the wound recovery phases in rats (infected with *C. albicans*) after beginning of curing (M ± Std. Dev.).

**Figure 3:**
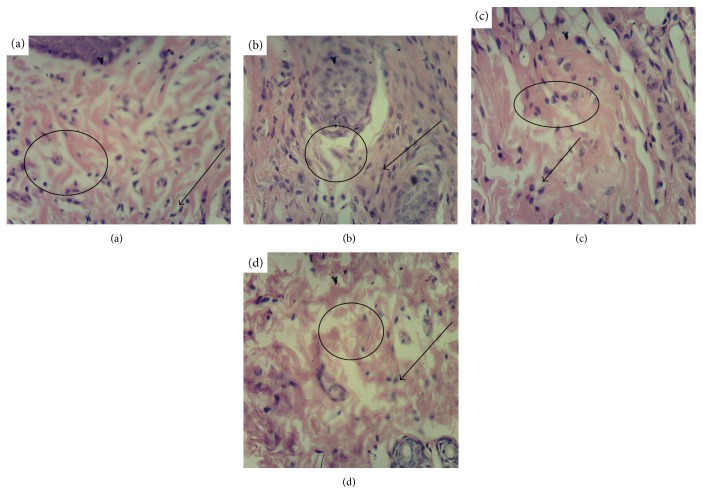
Histological analysis of derma in the wound bed, day 3 after beginning of curing (magnif. ×280). (a) Positive control (clotrimazole); (b) negative control (mock); (c) reference group (wild-type *K. pinnata*); (d) experimental group (CecP1). Selected area: circle/oval—edema in derma, arrow—leucocyte infiltration in the derma.

The wound healing process was divided into three partially overlapping stages: inflammation, granulation, and maturation. The stage of inflammation was considered to be finished once perifocal edema around the wound disappeared. Start of the granulation stage was registered once the wound bed began filling with a newly tissue of a pale pink color. Start of the maturation stage was registered once a marginal epithelialization became visible. Each animal was analyzed daily in order to reveal perifocal edema, granulation, and marginal epithelialization (Figure 1). Residual square of the wound was measured at 10th and 14th days after the beginning of curing (Figure 2). Significance of the difference between the groups is shown on Figure 3.

### 3.2. Histological Analysis of the Wound Healing Activity

Three days after the beginning of curing, the wound beds in all four groups were filled with purulent-necrotized cell mass (Figure 3). Leucocyte infiltration was visible deeply in the derma. An edema and plethora of dilated capillaries were found in the underlying tissues. The collagen fibers were disorganized and pushed away from each other due to interstitial edema.

**Figure 4:**
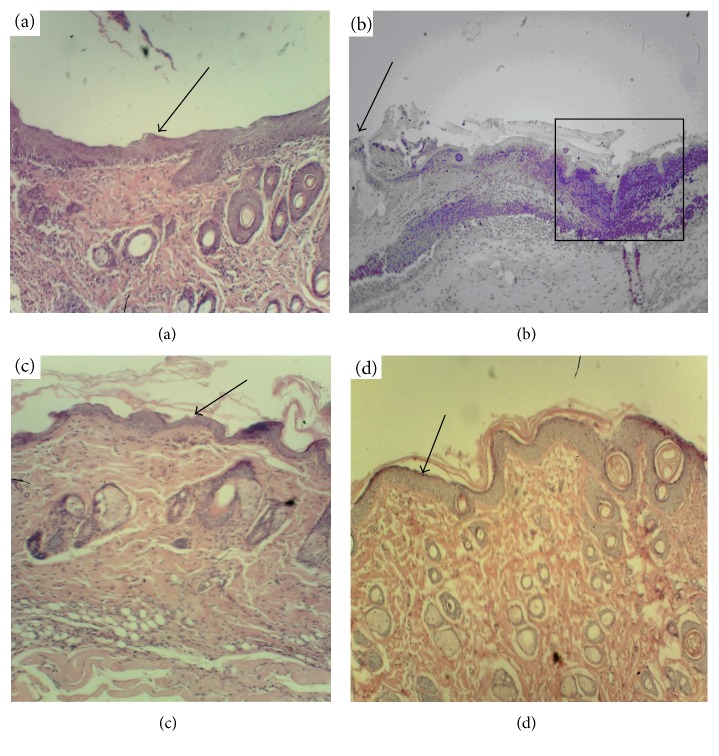
Histological analysis of derma within the wound bed, day 10 after the beginning of curing (magnif. ×70). (a) Positive control (clotrimazole); (b) negative control (no specific treatment); (c) reference group (wild-type *K. pinnata* extract); (d) experimental group (CecP1). Selected area: arrow—new-formed thin epithelium, square—area of melting of the necrotic tissue.

Ten days after the beginning of curing, the negative control group exhibited rising of the inflammation that was manifested as an appearance of a polynuclear inflammatory infiltrate, composed of neutrophils, macrophages, and must cells (Figure 4).

This period was characterized with melting of the necrotic tissue, their removal, and partial emptying of the wound cavity. The interstitial edema was conserved beneath the wound cavity indicating an increased permeability of the capillaries. Single microabscesses were found in deep layers of the derma. In the same period, noninfected animals did not exhibit traits of the inflammation. The surface of the wound was covered by separate islands of new-formed thin epithelium growing from the margins of the wound. Layers of derma beneath the wound were impregnated with fibrin and contained single fibroblasts. A wound bed in the positive control group (clotrimazole) contained granulation focuses constituted from inflammatory and proliferative cells and enriched with newly collagen fibers. A thin epithelial layer without abnormalities appeared along the margins of the wound. The reference group (extract of the wild-type *K. pinnata*) contained much more collagen fibers and fibroblasts beneath the edge of the wound then the negative and positive control groups. Marginal epithelialization was more pronounced in the reference group. However, the inflammatory cell component was more abundant than in the positive control group. The animals of the experimental group (CecP1) were characterized with the fastest progression in the collagen matrix formation, its optimal structuration, the fastest marginal epithelialization, and the minimal number of the polynuclear inflammatory infiltrate in the wound bed.

**Figure 5:**
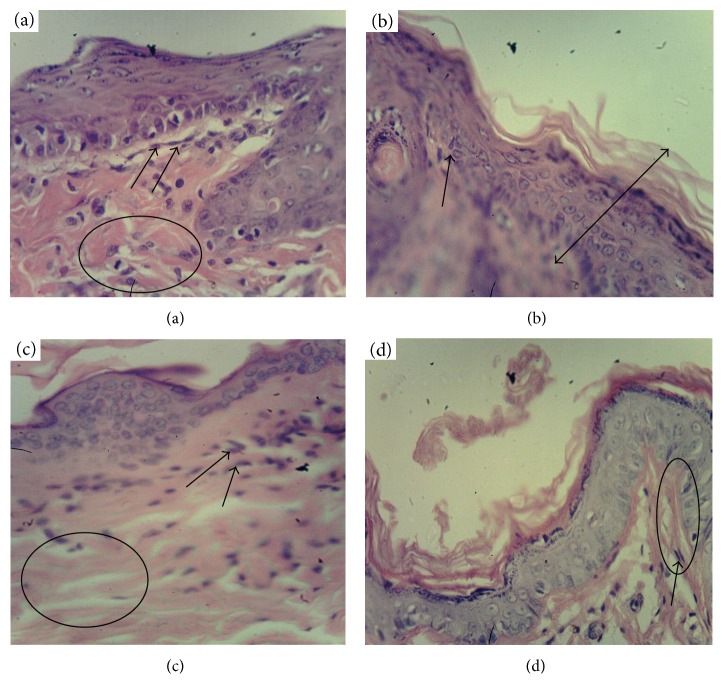
Histological analysis of derma in the wound bed, day 14 after the beginning of curing (magnif. ×280). (a) Positive control (clotrimazole); (b) negative control (no specific treatment); (c) reference group (wild-type *K. pinnata* extract); (d) experimental group (CecP1). Selected area: arrow—fibroblasts, circle—collagen fibers, and double arrow—thickened epidermis.

Fourteen days after the beginning of curing, the wound cavity of the negative control group was still filled with polynuclear inflammatory infiltrate (Figure 5).

Only single spindle-shaped fibroblasts were found. The new-formed granulation tissue was located in the bottom of the wound bed. The inflammatory infiltration and the interstitial edema were conserved in the depth of the derma. The singular thin collagen fibers were located irregularly. Most fibroblasts were clustered around them. A scar formation appeared as a solid layer of collagen fibers without vessels and skin appendages. The epidermis was much thicker than normal. The positive control group exhibited a similar picture although the collagen fibers were more abundant and less regular than in the negative control. The reference group (wild-type *K. pinnata* extract) did not contain solid collagen layer neither focuses; isolated inflammatory infiltrate focuses were conserved. However, overall structure of the collagen was more regular that in positive and negative control group. Revascularization of the scar was beginning. The experimental group combined positive traits of the positive control and the reference group. In this period, solid collagen layer completely covered the former wound cavity in this group. This layer was of the most regular, partially revascularized, and was completely free from the residual inflammation focuses.

### 3.3. Analysis of *C. albicans* Cell Survival In Vivo

**Figure 6:**
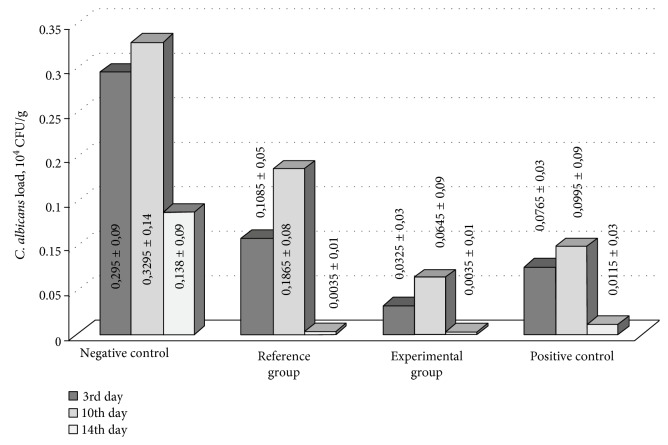
Dynamics of the wound bed contamination with *C. albicans.*

Fungicide activity of the tested medicines was evaluated as a decrease of alive *C. albicans* cell number per g of the granulate tissue determined by microbiological method as described in Method (Figure 6). An evident fungicidal activity of *K. pinnata* extract in vivo was found, and it was substantially increased by CecP1. The load of yeast cells in the granulate tissue at the 3rd day upon a treatment with the experimental preparation was even less than in the positive control (clotrimazole). In contrast, at the 10th day, the fungicide effect of clotrimazole was stronger than in the experimental *K. pinnata* extract with CecP1. In this period, distribution of the pathogen in the granulate tissue of the experimental group was much less homogeneous than the positive control group. At 14th day after the beginning of curing, *C. albicans* was eliminated over the most surface of the wound bed in the positive control and reference and experimental groups; however, single focuses of infection were conserved. In contrast, the infection evolved in the negative control group.

## 4. Conclusion

An evident fungicidal activity of *K. pinnata* against the most common human fungal pathogen *C. albicans* has been demonstrated for the first time. Moreover, the data of histological analysis make an evidence that *K. pinnata* exhibits a positive effect on remodeling of the collagen matrix and reepithelizaton of the wound cavity along with its repairing. As specified in Introduction, *K. pinnata* is known with the production of bufadienolides and polyphenols which exhibited a toxic activity of animal cells in vitro. However, our results give show that these compounds do not compromise wound healing activity when administrated within the water extracts of *K. pinnata* leaves normalized by the total protein load 1 mg/ml. CecP1 within the recombinant *K. pinnata* extract substantially ameliorates its natural antifungal properties making it comparable to the commonly used commercial antifungal preparation. In a certain extent, this result is unexpected, since many formerly studied AMP with *α*-helical secondary structure, on the one hand, exhibited unacceptable side-toxicity and, on the other hand, were unstable in vivo [34]. Contribution of immunomodulating activity of *K. pinnata* natural compounds to *C. albicans* elimination in vivo is confirmed by the fact that although CecP1 exhibits 80–300-fold lower molar activity against *C. albicans* in vitro than clotrimazole, CecP1-enriched extract of *K. pinnata* provides even more fast and complete elimination of the fungal pathogen from the infected wound than the commercial fungicide. Taken together, our data demonstrate a high perspectivity of pharmaceutical application of *K. pinnata* extracts containing CecP1 for curing *C. albicans* infection and possibly other fungal infections. This approach is technologically and economically affordable. However, an extensive toxicological trials and testing of rising resistance mechanisms in the fungal pathogens against the novel potential drug are required before it can be recommended for a practical implementation.
